# Increased Inflammatory Signaling and Lethality of Influenza H1N1 by Nuclear Thioredoxin-1

**DOI:** 10.1371/journal.pone.0018918

**Published:** 2011-04-15

**Authors:** Young-Mi Go, Sang-Moo Kang, James R. Roede, Michael Orr, Dean P. Jones

**Affiliations:** 1 Division of Pulmonary, Allergy and Critical Care Medicine, Department of Medicine, Emory University, Atlanta, Georgia, United States of America; 2 Microbiology and Immunology, Emory University, Atlanta, Georgia, United States of America; Cleveland Clinic, United States of America

## Abstract

**Background:**

Cell culture studies show that the antioxidant thiol protein, thioredoxin-1 (Trx1), translocates to cell nuclei during stress, facilitates DNA binding of transcription factors NF-κB and glucocorticoid receptor (GR) and potentiates signaling in immune cells. Excessive proinflammatory signaling *in vivo* contributes to immune hyper-responsiveness and disease severity, but no studies have addressed whether nuclear Trx1 mediates such responses.

**Methodology/Principal Findings:**

Transgenic mice (Tg) expressing human Trx1 (hTrx1) with added nuclear localization signal (NLS) showed broad tissue expression and nuclear localization. The role of nuclear Trx1 in inflammatory signaling was examined in Tg and wild-type (WT) mice following infection with influenza (H1N1) virus. Results showed that Tg mice had earlier and more extensive NF-κB activation, increased TNF-α and IL-6 expression, greater weight loss, slower recovery and increased mortality compared to WT. Decreased plasma glutathione (GSH) and oxidized plasma GSH/GSSG redox potential (E_h_GSSG) following infection in Tg mice showed that the increased nuclear thiol antioxidant caused a paradoxical downstream oxidative stress. An independent test of this nuclear reductive stress showed that glucocorticoid-induced thymocyte apoptosis was increased by NLS-Trx1.

**Conclusion/Significance:**

Increased Trx1 in cell nuclei can increase severity of disease responses by potentiation of redox-sensitive transcription factor activation.

## Introduction

Thioredoxin-1 (Trx-1) and glutathione (GSH) are central thiol redox systems in cell nuclei and cytoplasm but are differentially regulated in these compartments. For instance, during cell stress induced by nutrient deprivation, proinflammatory signals, oxidants or reactive electrophiles, the nuclear pools are more resistant to oxidation or depletion [1], [2], [3]. Although little is known about the adaptive mechanisms for the nuclear GSH system, the function of the nuclear Trx1 system is enhanced by translocation of Trx1 from cytoplasm to nuclei. This translocation is critical to function of transcription factors that contain a regulatory cysteine in the DNA binding region, including nuclear factor-κB (NF-κB) [4], [5], [6], glucocorticoid receptor (GR) [7], [8], activator protein-1 [9], [10], [11], nuclear factor (erythroid-derived 2)-like 2 (Nrf-2) [12], [13], Hypoxia-inducible factor 1 alpha (HIF-1α) [14] and p53 [15], [16].

Compartmental regulation of NF-κB, AP-1 and Nrf-2 involves opposing redox-sensitive steps in cytoplasm and nuclei, i.e., 1) upstream cytoplasmic oxidative activation involving kinase signaling and 2) downstream Trx1-dependent reduction of the Cys of the DNA-binding domain [9], [17], [18], [19]. For example, oxidative signaling in the cytoplasm initiates NF-κB activation via I-κB kinase, which phosphorylates I-κB causing dissociation and release of NF-κB for translocation into the nucleus [20]. Excessive oxidant production oxidizes a critical Cys^62^ in the DNA binding region of NF-κB and inhibits DNA binding [6], [18]. Increased nuclear Trx1 by transient transfection enhances DNA binding and increases NF-κB reporter activity [5]. Studies with targeted increases in peroxiredoxin (Prx)-1 further suggest that the nuclear activation by Trx1 counters an endogenous H_2_O_2_-dependent transcriptional termination mechanism [21].

Modulation of NF-κB signaling by nuclear Trx1 raises the possibility that excessive nuclear Trx1 could cause hyper-responsive immune signaling. NF-κB activation is induced by viruses and viral products, including influenza and HIV, as well as other stimuli associated with oxidative stress (free radicals, UV light, gamma-irradiation) [22]. NF-κB modulates the induction of multiple proinflammatory cytokines, including interleukin (IL)-1β, IL-6 and tumor necrosis factor (TNF)-α, and is also induced by these proinflammatory cytokines [22], so that excessive NF-κB activity due to inadequate nuclear inactivation could contribute to severity of immune-mediated disease symptoms. Excessive activation of the immune system has been linked to severity of infection and death due to H1N1 influenza viral infection [23], [24], [25].

Cytoplasmic redox regulation of GR differs from NF-κB, but increased nuclear Trx1 could similarly contribute to excessive transcriptional activity. GR is a transcription factor belonging to a family of nuclear receptors [7], [26], [27] with different redox-sensitive steps in the compartments. Unlike the cytoplasmic oxidative activation described above, GR must be reduced to bind ligand and be transported into nuclei. During oxidative stress, the Trx1 system in the cytoplasm [7], [26], [27], [28] in combination with the mitochondrial Trx2 system [29], preserve GR function by protecting against oxidation. In nuclei, Trx1 directly interacts with GR through DNA and ligand-binding domains, and maintains GR in a reduced, active transcriptional state [7], [27]. Activation of GR by binding of glucocorticoid hormones (GH) regulates T-cell survival in positive and negative selection of the immune system by regulating apoptosis of thymocytes and T lymphocytes [30], [31]. Excessive activation of GR could therefore contribute to impaired immune functions [32].

Many studies show that oxidative inactivation can disrupt regulation of transcription and contribute to disease [33], [34], but relatively little information is available concerning the possible contribution of excessive nuclear reduction. If excessive nuclear reduction contributed to severity of H1N1 influenza, therapeutic strategies targeting nuclear Trx1 may prove useful to protect individuals severely affected because of immune-mediated injury. To test the possibility that excessive nuclear reduction could contribute to hyper-responsive immune signaling, we created a transgenic mouse model (NLS-hTrx1 Tg) in which nuclear Trx1 was increased due to expression of a fusion protein containing human Trx1 with a nuclear localization signal (NLS). Using this *in vivo* model, we show that NF-κB activity is controlled by nuclear Trx1. Tg mice with H1N1 influenza infection had greater inflammatory response, including elevated NF-κB activity and IL-6 and TNF-α induction. This increased response was associated with impaired body weight recovery and increased mortality due to the viral infection. In an independent test of the regulatory function of nuclear Trx1, we observed that increased nuclear Trx1 also potentiated glucocorticoid-stimulated thymocyte death in the transgenic model. Together, the data show that increased nuclear Trx1 creates a type of reductive stress in cell nuclei that could contribute to a hyper-responsive immune activation and represent a potential target for therapeutic intervention.

## Materials and Methods

### Ethics Statement

All protocols involving mice were reviewed and approved by the Institutional Animal Care and Use Committee at Emory University.

### Wild-type and NLS-hTrx1 transgenic mouse

All animal experiments and husbandry for the studies presented in this manuscript were conducted under the review and approval of the Emory University Institutional Animal Care and Use Committee (IACUC, approval ID: DAR-2000040-062113). Emory IACUC operates under the federal Animal Welfare Law (administered by the USDA) and regulations of the Department of Health and Human Services. Mice were maintained in the Emory University Division of Animal Resources Facility under a standard 12-h light/dark cycle and had free access to tap water and standard laboratory diet. C57BL/6 (WT) mice were purchased from Charles River Laboratories and used to maintain the transgenic mouse colony, with WT mice in experiments being littermates of transgenic mice (Tg) expressing the human Trx1 (hTrx1) gene. The hTrx1 was modified to contain c-Myc epitope tag at the N-terminus and nuclear localization signal (NLS) at the C-terminus (defined as NLS-hTrx1 Tg). Preliminary studies using transient transfection showed that NLS-Trx1 constructs with 1 or 3 copies of the NLS and a Myc epitope tag were expressed in cells in culture and showed expected activity in experiments with an NF-κB reporter assay. NLS-Trx1 constructs were used by the Emory University Transgenic Mouse Core to create a total of 6 transgenic lines. Briefly, Trx1 cDNA was inserted between the cytomegalovirus (CMV) promoter and the simian virus (SV-40) terminator of pCMV-Myc mammalian expression vector purchased from Clontech Laboratories (Mountain View, CA). The transgenic construct was microinjected into the pronucleus of fertilized eggs from C57BL/6 mice. The presence of the NLS-hTrx1 transgene was confirmed by PCR analysis using mouse genomic DNA prepared from tail biopsy as a template and the following oligonucleotide primers: forward primer, 5′-ATG GCA TCA ATG CAG AAG CTG ATC T-3′; and reverse primer, 5′-GCC GCT GGA TCT TCT ACC TTT CTC T-3′ (Integrated DNA technologies, Iowa). Three mouse lines were created with a single NLS sequence: these showed mRNA expression for NLS-hTrx1 but no detectable cellular or nuclear Myc by western blot or fluorescence immunohistochemistry. Consequently, additional lines were created with 3 tandem copies of the NLS sequence. These mice appeared phenotypically normal in terms of weight, activity and appearance, and one line was selected for study.

### Quantification of NLS-hTrx1 and cytokines by real-time PCR

Total tissue mRNA was isolated using RNeasy Mini kit purchased from Qiagen (Valencia, CA) following the manufacturer's protocol, and reverse transcription was performed to generate cDNA (Clontech Laboratories, Mountain View, CA). For quantitative real-time PCR, amplification was performed on an iCycler IQ Multicolor Real-Time PCR Detection System (Bio-Rad Laboratories, Hercules, CA) for 35 cycles as follows: 95°C for 30 seconds, 58°C for 30 seconds, and 72°C for 1 min. Quantification and melting curves were analyzed with iCycler software. Primers for cytokines were designed using a program provided by Integrated DNA Technologies (Coralville, Iowa). Details of PCR primer sequences and product sizes for mouse cytokines used in the analyses of extracted RNA for quantitative real-time PCR are as follows; IL-1β (180 bp), forward: 5′-TGGAGAGTGTGGATCCCAAGCAAT-3′, reverse: 5′-TGTCCTGACCACTGTTGTTTCCCA-3′; IL-6 (134 bp), forward: 5′-ATCCAGTTGCCTTCTTGGGACTGA-3′, reverse: 5′- TAAGCCTCCGACTTGTGAAGTGGT-3′; IL-10 (147 bp), forward: 5′- TGAATTCCCTGGGTGAGAAGCTGA-3′, reverse: 5′- TGGCCTTGTAGACACCTTGGTCTT-3′; TNFα (140 bp), forward: 5′-ACGGCATGGATCTCAAAGACAACC-3′, reverse: 5′- TGAGATAGCAAATCGGCTGACGGT-3′; CSF (154 bp), forward: 5′- TGGCTTGGCTTGGGATGATTCTCA-3′, reverse: 5′- CCCATGGTTTGGTTGCTCTGTTGA-3′.

### Subcellular fractionation and western blotting

Subcellular fractionation of lung and kidney tissues was performed using Qproteome kit (Qiagen, Valencia, CA) following the procedures provided by the manufacturer. These organ systems were examined because of the expected sensitivity of lung to influenza virus and because of the abundance of NLS-Trx1 in the kidney. Twenty mg of kidney and lung from WT and Tg were processed to isolate cytoplasmic and nuclear fractions. Isolated fractions were then confirmed by western blotting probed with antibodies (ab) against GAPDH and lamin B for cytoplasm and nuclei, respectively. To examine abundance of other antioxidant proteins by western blotting, antibodies used were as follows: Myc (Cell Signaling Technology, Danvers, MA); hTrx1 and mTrx1 (AbFrontier, Seoul, Korea); Trx2 (antisera produced by Covance, Princeton, NJ); Prx1, Prx2 and Prx3 (Abcam, Cambridge, MA); Txnip (Invitrogen, Carlsbad, CA); actin (Sigma-Aldrich, St. Louis, MO); Alexa-Fluor-680-conjugated anti-rabbit or anti-mouse secondary antibody (Invitrogen). A band corresponding to each protein was visualized using an Odyssey scanner and Odyssey 2.1 software (Li-Cor, Lincoln, NE).

### Nuclear localization by immunohistochemistry and fluorescence microscopy

Cells isolated from WT and Tg kidneys were plated on glass cover slips. After 1 d, cells were washed, fixed, stained with anti-Myc ab followed by Cy3-conjugated goat anti-rabbit ab (Jackson Immuno Research, West Grove, PA) to visualize nuclear localization of NLS-hTrx1. Alexa Fluor 488 phalloidin was used to stain actin. Immunofluorescence was visualized using an Olympus X-70 fluorescence microscope system.

### DNA binding activity measurement by electrophoretic mobility shift assay (EMSA)

Nuclear extracts prepared from lungs of Tg and WT mice prior to and 3 d post infection were analyzed using an EMSA kit (Affymetrics, Clara, CA) by incubating a biotin-labeled or unlabeled probe containing an NF-κB DNA-binding consensus sequence (5′-AGTTGAGGGGACTTTCCCAGGC-3′) with a nuclear extract (5 µg) without added reductant for 30 min at 15°C. The samples were analyzed on a 6% nondenaturing PAGE and electroblotted for 30 min. Signals were detected by chemiluminescence imaging according to the manufacturer's protocol.

### Transfection and NF-κB luciferase activity measurement

HeLa cells purchased from ATCC were maintained with 10% FBS (37°C, 5% CO_2_) in DMEM. Cells at 80% confluence were co-transfected with pcDNA3.1, Trx1 WT, or Trx1 C35S [35], and with pNF-κB Luc (BD science, Franklin Lakes, NJ) plus pcDNA3.1 containing lacZ gene (Invitrogen) using lipofectamin (Invitrogen). Two days after transfection, luciferase activity was measured and normalized for transfection efficiency by β-galactosidase activity. To initiate luciferase activity, cell lysates (20 µl) were added to 100 µl to reaction buffer (Promega, Madison, WI) and luminescence was recorded at 30°C using a luminometer. β-galactosidase activity was quantified by monitoring cleavage of *o*-nitrophenyl-β-d-galactopyranoside.

### Glutathione (GSH), cysteine (Cys) and related measurements

Plasma and lung tissue collected from Tg and WT mice 3 d after infection or with no infection were analyzed for GSH, glutathione disulfide (GSSG), Cys, and cystine (CySS) by high performance liquid chromatography (HPLC) with fluorescence detection [36]. Values were used to calculate the steady-state redox potential for GSH/GSSG (E_h_GSSG) and Cys/CySS (E_h_CySS) using the measured concentrations, the Nernst equation, and respective E_o_GSSG and E_o_CySS at pH 7.4 for plasma (−264 mV, −250 mV) and 7.0 for lung (−240 mV, −226 mV) [36], [37].

### Influenza virus infection

H1N1 influenza virus (A/California/04/2009) was kindly provided by Dr. Richard Webby. The virus was grown in 11-d old embryonated hen's eggs for 2 d at 37°C, harvested and stored at −80°C until use. Mice were infected with serial dilutions of A/California/04/2009 virus and the 50% lethal dose (LD_50_) was determined as described [38].

Wild-type and NLS-hTrx1 transgenic mice (8 to 10 weeks) were used for infection. Isoflurane-anesthetized mice (18 WT, 17 Tg) were intranasally infected with 1× LD_50_ of A/California/04/2009 virus (1× the 50% lethal dose, LD_50_) in 50 µl of phosphate-buffered saline (PBS). Mice were observed daily to monitor changes in body weight and to record mortality (25% loss in body weight as the Institutional Animal Care and Use Committee (IACUC) endpoint for morbidity). For the analysis of lungs at early time points, additional groups of mice (6 mice for Tg, 5 mice for WT) were infected and euthanized at 2- or 3-d post infection to collect lung samples.

### Isolation of thymocytes and glucocorticoid-induced cell death

Thymocytes, from 4 to 6 week-old Tg and WT mice, were enriched by passage through nylon wool columns. The effects of glucocorticoid (dexamethasone, Sigma-Aldrich Corp, St. Louis, MO)-induced apoptosis were evaluated. Cell survival and death were quantified by measuring absorbance of the dye product from the nonradioactive quantitative reagent WST-1 (Roche, Basel, Switzerland) and by cell counts using a hemocytometer.

### Statistics

Statistical comparisons of data were carried out using the t-test of the OriginLab (Data Analysis and Graphing software, OriginLab Co.). P<0.05 was considered to be significant.

## Results

### Characteristics of NLS-hTrx1 Tg Mice

**Figure 1:**
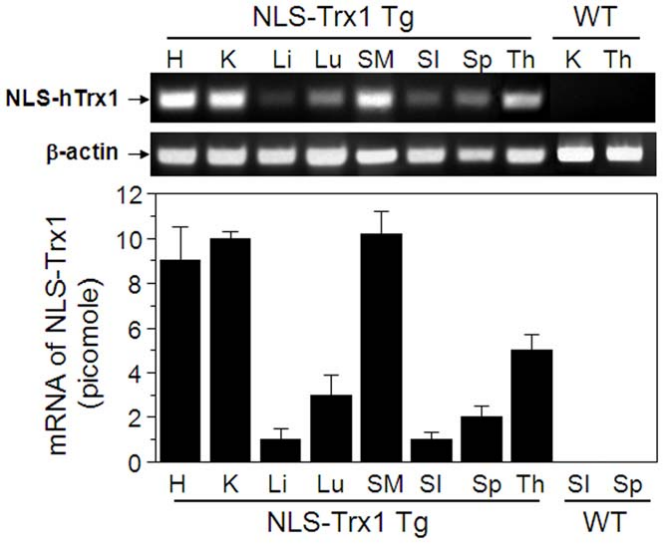
mRNA levels of NLS-hTrx1 in different tissues. Different organs harvested from Tg and littermate WT mice were analyzed to examine mRNA abundance of nuclear compartment-targeted hTrx1 and β-actin. Each cDNA converted from purified tissue RNA (1 µg) by reverse transcription was analyzed by real-time PCR. Quantified values (picomole) are represented as mean ± SE for 4 Tg and 4 WT mice. Symbols are as follows; H, heart; K, kidney; Li, liver; Lu, lung; SM, skeletal muscle; SI, small intestine; Sp, spleen; Th, thymus.

To examine tissue distribution of NLS-hTrx1 mRNA, cDNA converted from total RNA isolated from tissues of littermates with transgene-positive and -negative genotypes. Results showed greater abundance of mRNA for NLS-hTrx1 in heart, kidney, skeletal muscle, and thymus, and lower amounts in liver, lung, small intestine, and spleen (Fig. 1). Thus, the NLS-Trx1 transgene is expressed in multiple organ systems as expected based upon the promoter used.

**Figure 2:**
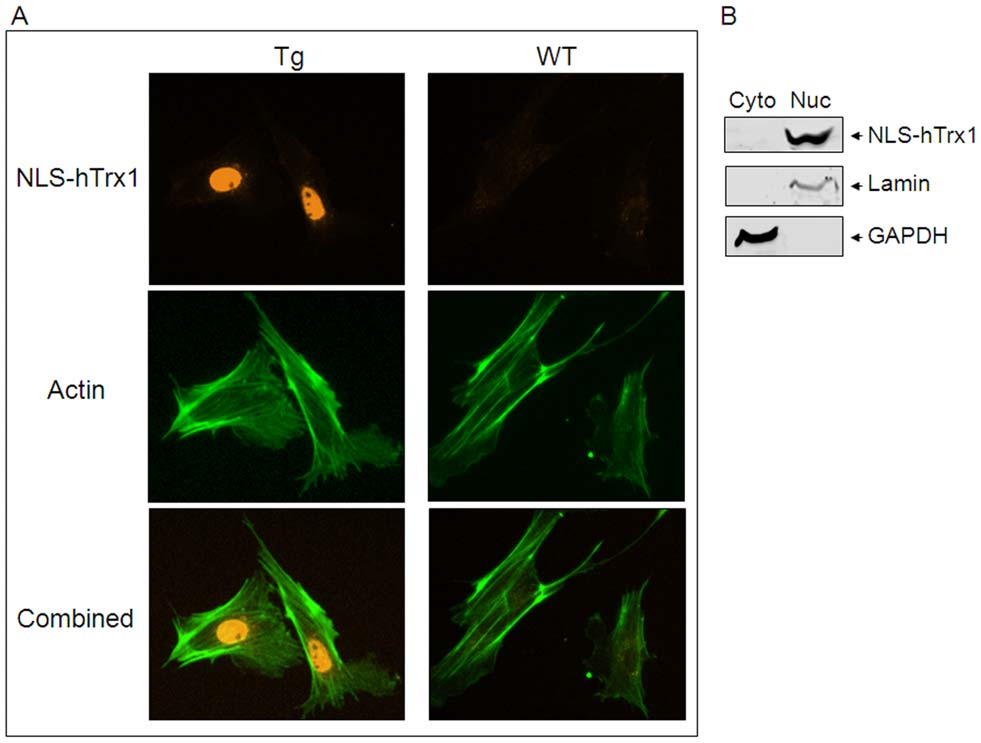
Nuclear localization of NLS-hTrx1 in Tg mouse. Cells isolated from kidneys of Tg and WT were analyzed to examine localization by immunofluorescence (A) and western blotting (B). A, Nuclear compartmentalized expression of NLS-hTrx1 was visualized by myc antibody followed by Cy3 (orange, Tg). As a control, actin distribution in all area of cells was obtained by Alexa Fluor 488 phalloidin, shown for both Tg and WT (green). Subcellular fractions including cytoplasm and nuclei were obtained from kidney tissues of Tg and WT. Western blotting using each fraction confirmed nuclear localization of NLS-hTrx1 in Tg. Western blot analysis of lamin and GAPDH were used as markers to verify purity of nuclear and cytoplasmic fractions, respectively.

Characterization of the abundance and localization of the NLS-hTrx1 transgene product was facilitated by inclusion of the c-Myc epitope, which allowed discrimination from the endogenous mouse Trx1. Subcellular localization studies were performed on kidney because of the relative abundance of the transgenic product in kidney, the ability to obtain adherent cells for fluorescence microscopy and the ability to obtain quality nuclear preparations. Immunofluorescence studies of cells isolated from kidney (Fig. 2A) using anti-Myc antibody with cy3 (orange), showed NLS-hTrx1 present in cell nuclei of transgenic (Tg) mice (Fig. 2A, top). In contrast to Tg, WT shows no detection of NLS-hTrx1 in nuclei while actin was detected in both Tg and WT (Fig. 2A, middle).

Western blot analysis of cytosolic and nuclear fractions of kidney (Fig. 2B) confirmed these results. Immunoblots of cytoplasmic and nuclear fractions of kidney tissue obtained from Tg mice shows nuclear localization of NLS-hTrx1 detected using anti-Myc antibody (Fig. 2B). Extracts from kidneys of wild-type mice showed no reactivity. The same blots re-probed with antibodies to marker proteins, lamin (nuclei) and GAPDH (cytosol), confirmed the purity of the subcellular fractions. Together, the data show that the hTrx1 protein is produced and localized to cell nuclei in this NLS-hTrx1 transgenic mouse model.

**Figure 3:**
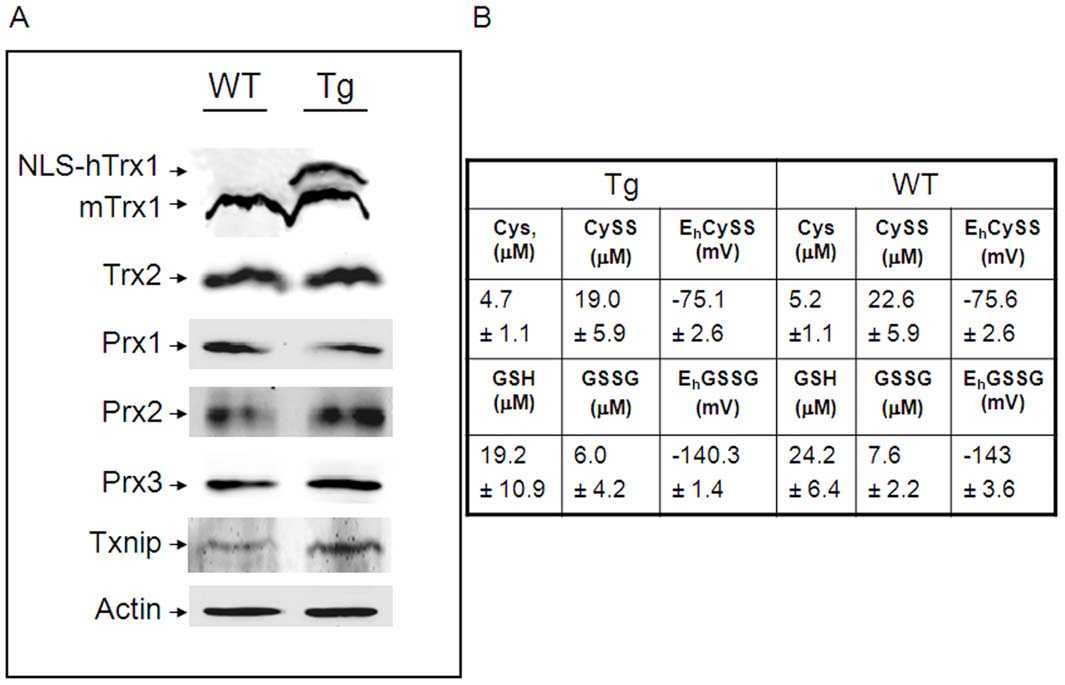
The effect of NLS-hTrx1 on abundance of antioxidant proteins and plasma redox potentials. Protein abundance was examined by western blot analysis with antibodies specific to Trx1 (NLS-hTrx1 and mTrx1), Prx1, Prx2, Prx3, Txnip, and actin (A). Western blots show representative data of kidney tissues obtained from 3 Tg mice and littermate WT controls. B, Plasma base line concentrations of cysteine (Cys), cystine (CySS), glutathione (GSH), and glutathione disulfide (GSSG) in Tg and WT mice were determined by HPLC analyses. Redox potentials of Cys/CySS (E_h_CySS) and GSH/GSSG (E_h_GSSG) were calculated by using the Nernst equation [60].

We next examined whether expression of NLS-hTrx1 altered the abundance of other antioxidant proteins including mTrx1, mitochondrial Trx2, nuclear and cytoplasmic peroxiredoxins (Prx1, Prx2), mitochondrial Prx3, and thioredoxin-interacting protein (Txnip). Expression of hTrx1 had no effect on the abundance of mouse Trx1 but approximately doubled the total amount of Trx1 (Fig. 3, Top panel). There appeared to be a slightly higher Prx2 and Txnip in some Tg mice (Fig. 3A) but this was not significant.

To determine whether increased nuclear Trx1 had a systemic effect on redox state, we analyzed the redox potentials (E_h_) of GSH/GSSG and Cys/CySS in plasma. Results show that there were no significant differences between WT and Tg mice in concentrations of Cys, CySS, GSH, and GSSG or E_h_CySS and E_h_GSSG (Fig. 3B). Together, the data show that the Tg mice have increased nuclear Trx1 and that this increase does not have major effects on other thiol antioxidant components or gross phenotype.

### Trx1 over-expression in cell nuclei potentiates viral infection-induced weight loss and mortality

**Figure 4:**
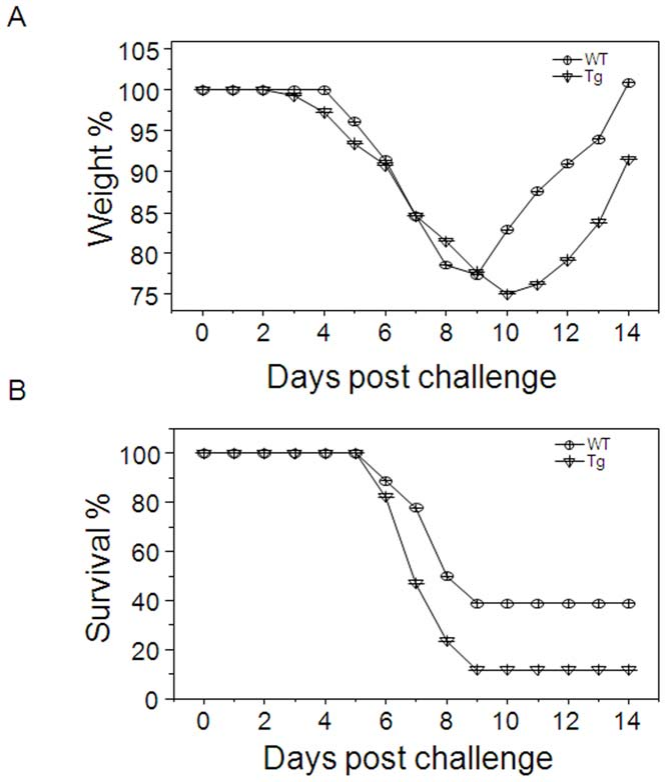
Effect of influenza virus infection on (A) body weight and (B) survival in NLS-hTrx1 Tg mice compared to WT littermates. WT (n = 18) and Tg mice (n = 17) were infected with 2009 H1N1 influenza A/California/04/2009 virus (1× LD50) as described previously [38]. Body weight and survival rates (WT; 7/18, Tg; 2/17) were monitored daily and presented as percentages based on day 0.

The 2009 H1N1 influenza virus (A/California/2009) administered at the LD_50_ was pathogenic to both the wild-type and NLS-hTrx1 transgenic mice as shown by body weight loss (Fig. 4A). The WT mice showed symptoms of illness, including weight loss, starting at 4 or 5-d post infection, while the Tg mice showed weight loss at 3 to 4 d (Fig. 4A). From 6 to 9-d post infection, both groups of mice suffered severe loss in body weight. The WT mice had a survival rate of 40% (WT; 7/18 mice, Fig. 4B). The Tg mice showed only 11.8% survival (Tg; 2/17 mice, Fig. 4B), and morbidity occurred earlier than in WT. Also, the recovery of Tg mice from influenza virus infection was delayed compared to WT mice. These results show that NLS-hTrx1 Tg mice had more severe disease response to viral infection and did not recover as well.

### NF-κB activation and cytokine expression in Tg mice infected with influenza

**Figure 5:**
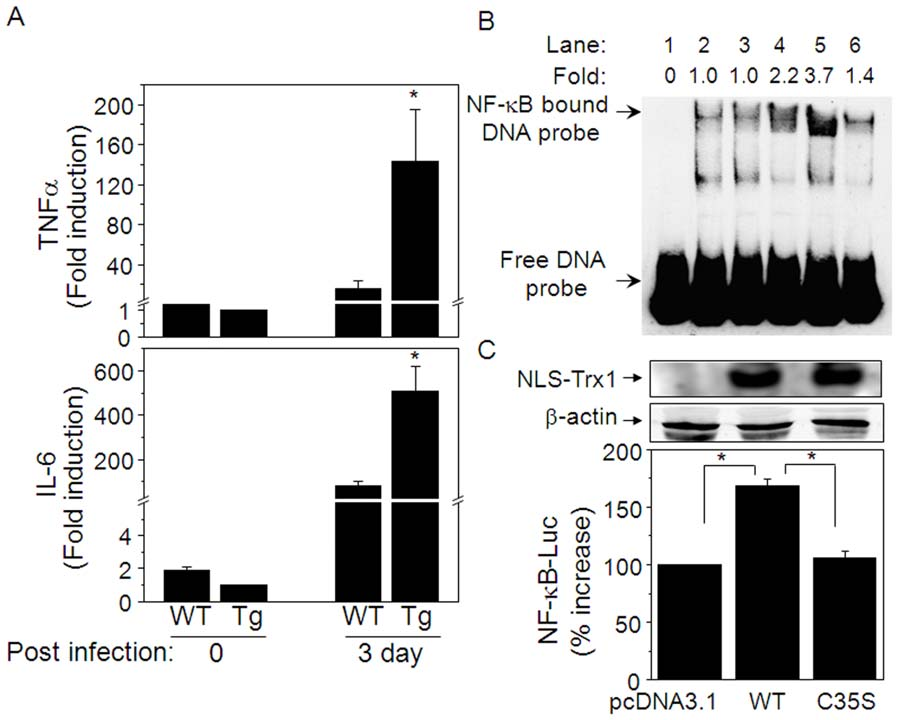
NLS-hTrx1 transgenic mice have increased NF-κB activation and proinflammatory cytokine (TNF-α and IL-6) production after influenza viral infection. Lung tissues obtained from Tg and WT before (3 mice each for Tg and WT) and 3-d post infection (6 mice for Tg, 5 mice for WT) were examined for mRNA of cytokines (A) and NF-κB activity (B). mRNA levels of TNF-α and IL-6 were analyzed and quantified by real-time PCR. * p<0.05 for 3-d post infection in Tg compared to 3 d post infection in WT and 0 d in Tg. B, NF-κB activity was examined by EMSA using same tissues analyzed for cytokines shown in A. Bands indicated as NF-κB bound DNA probe show activity of NF-κB. Densitometry values shown as fold difference were obtained from measuring relative intensities compared to that in WT before infection (lane 2). [lanes 1–6 are as follows; 1, NF-κB probe alone; 2, WT before infection; 3, Tg before infection; 4, WT 3 days post infection; 5, Tg 3-d post infection; 6, Tg 3-d post infection (cold NF-κB probe incubation followed by labeled NF-κB probe incubation)]. C, NF-κB activity (NF-κB Luc) was examined by expression of NLS-hTrx1 WT or dominant negative mutant of Trx1, NLS-hTrx1 C35S by transient cotransfection with NF-κB luciferase and β-galactosidase. Quantified luciferase activity as a measure of NF-κB activity was normalized by β-galactosidase [37]. Western blot confirms expression of NLS-hTrx1 WT and NLS-hTrx1 C35S (C, top) and β-actin (C, middle) for equal protein loading in HeLa cells 2 d after transfection. Results of luciferase activity in bar graph are shown as means ± SE (n = 8, * p<0.05).

To test whether increased mortality and poor recovery of NLS-hTrx1 Tg following viral infection was associated with increased amounts of inflammatory cytokines, we analyzed mRNA levels of colony stimulating factor (CSF), IL-1β, IL-6, IL-10, and TNFα in lung tissues of infected mice at 3-d post infection. Both TNFα (Tg, 143.5±51.5; WT, 16.0±7.3, Fig. 5A top) and IL-6 (Tg, 509.3±109.2; WT, 79.7±21.2, Fig. 5A bottom) were significantly elevated 3-d after H1N1 infection in WT and Tg mice relative to untreated controls [TNFα (Tg; 1.0, WT; 1.3±0.3), IL-6 (Tg; 1.0, WT; 1.9±0.2)]. However, the magnitude of the induction for both (9.0-fold for TNFα and 6.3-fold for IL-6) was much greater in Tg than WT. There were no significant differences in mRNA levels for CSF, IL-1β and IL-10 (data not shown).

IL-6 and TNFα are expressed under the control of the transcription factor NF-κB. To determine if DNA-binding activity was increased in Tg mice, we analyzed nuclear extracts obtained from WT and Tg lungs before [lanes 2 (WT) and 3 (Tg) in Fig. 5B] and 3-d post infection [lanes 4 (WT), 5 (Tg) in Fig. 5B] using an EMSA without added reductant. The results showed that NF-κB binding was activated 3-d post infection (Fig. 5B, lane 5: Tg) and an increased level of activation was present in Tg (lane 5) than WT (lane 6). The same sample used for lane 5 (Tg, 3-d post infection) was incubated with unlabeled NF-κB probe before incubation with labeled NF-κB probe to test specific interaction between NF-κB and DNA probe (lane 6). The results showed no intensity of NF-κB-bound labeled probe due to competition between unlabeled- and labeled NF-κB probes suggesting that NF-κB binding to the DNA probe is specific. Mean activities of NF-κB (Fig. 5B) for each lane with respective activity expressed as fold increase relative to pre-infection WT control (Lane 2) are as follows: 1, 0; 2, 1.0; 3, 1.0; 4, 2.2; 5, 3.7; 6, 1.4.

Since Trx1 regulates NF-κB activation in a redox-dependent manner, we examined whether increased activation of NF-κB in NLS-hTrx1 Tg was regulated by Trx1 through its catalytic redox activity. To address this question, we used an *in vitro*, transient transfection model because a transgenic mouse with a dominant negative mutant of NLS-hTrx1 is not available. Fig. 5C shows that NF-κB luciferase activity was elevated by NLS-hTrx1 WT (vector corresponding to that used to make NLS-hTrx1 Tg mouse) but not by a dominant negative form, NLS- C35S -hTrx1, supporting previous data that redox-active Trx1 in cell nuclei controls NF-κB activation by a redox-dependent mechanism.

**Figure 6:**
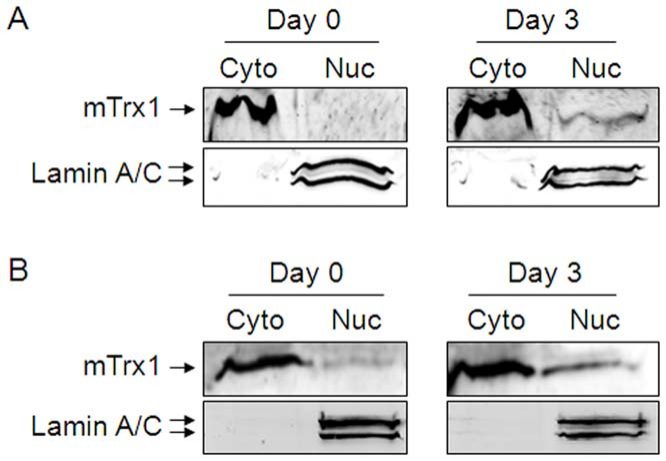
Increased endogenous mouse Trx1 level in nuclei by infection with influenza H1N1 virus. WT mice infected with influenza H1N1 virus (A/California/04/2009) at day 3 post infection (day 3) or uninfected WT control mice (day 0) were examined for nuclear Trx1. Cytoplasm (Cyto) and nuclear (Nuc) fractions of lung tissues (A) and immune cells isolated from lungs were examined for Trx1 level by western blot analysis. The same blot was probed with a lamin A/C antibody as a nuclear protein marker. Results are representatives of 3 analyses.

Influenza infection is associated with oxidative stress in the lungs. Since previous findings have shown nuclear translocation of Trx1 under oxidative stress conditions [39], [40], we examined whether infection with H1N1 influenza virus affected endogenous levels of Trx1 in lung cell nuclei in WT mice. Results show that there were small increases in endogenous mouse Trx1 (mTrx1) levels in nuclei isolated from the lung tissues (Fig. 6A, Day 3) and immune cells (Fig. 6B, Day 3) of infected mice.

### Plasma E_h_GSSG is oxidized to a greater extent in Tg mice with viral infection

**Figure 7:**
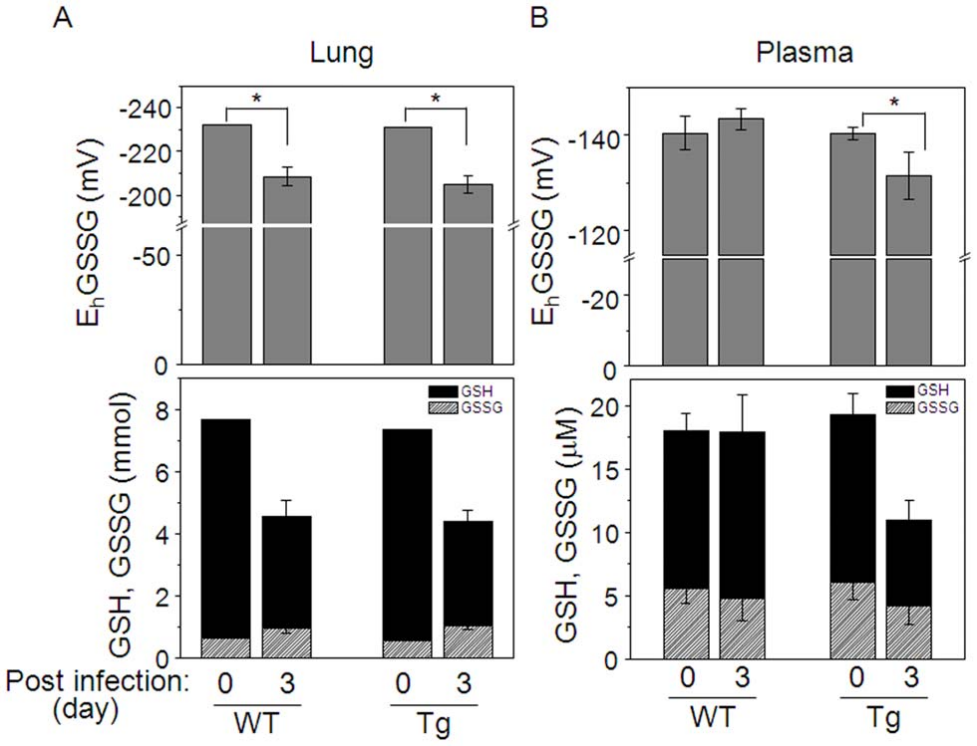
Changes in GSH/GSSG redox potential (E_h_GSSG) in Tg and WT following H1N1 influenza virus infection. The same lung samples described in Fig. 5 were examined for GSH and GSSG concentrations by HPLC, and E_h_GSSG was calculated using the Nernst equation [60]. No differences in concentrations or E_h_GSSG oxidation were observed in lung tissues of Tg or WT following infection, but more oxidation of plasma E_h_GSSG was observed in Tg compared to WT. Three mice for each Tg and WT without infection were analyzed as control (0 in A). Plasma GSH, GSSG, and E_h_GSSG in Tg and WT before and 3 d post infection are shown in B. * p<0.05.

To determine whether increased nuclear Trx1 protected against oxidative stress following influenza infection, we measured GSH, GSSG, Cys and CySS and calculated E_h_GSSG and E_h_CySS redox potentials in lung tissue and plasma before and 3-d post infection in WT and Tg mice. Lung E_h_GSSG of both WT and Tg was significantly oxidized after infection, but there was no statistical difference between WT and Tg. Lung tissue E_h_CySS was not measurable due to low levels of Cys and CySS in tissue. In WT, viral infection had no significant effect on plasma GSH or plasma E_h_GSSG. However, plasma GSH was decreased in Tg after infection [Fig. 7A bottom: Tg (0 d), 19.2±1.7 µM; Tg (3 d); 10.9±1.6 µM] and E_h_GSSG was more oxidized [Fig. 7A top: Tg (0 d), −140±1.4 mV; Tg (3 d), −131. 5±4.8 mV]. E_h_CySS was unchanged in Tg and WT at 3-d post infection (data not shown).

### Nuclear Trx1 substantiates glucocorticoid-stimulated apoptosis

**Figure 8:**
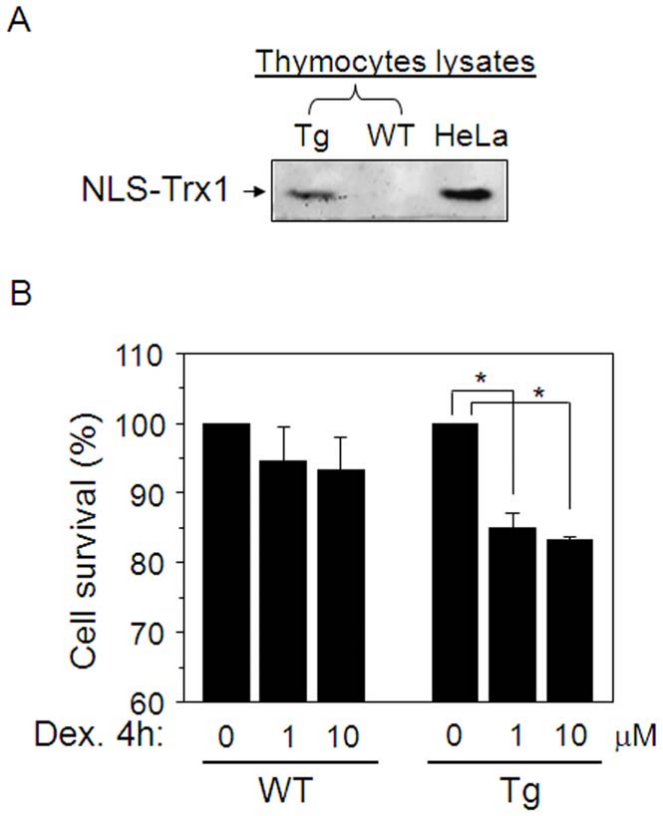
Trx1 in nuclei stimulates dexamethasone-induced death in thymocytes from immature mice. Western blot shows NLS-hTrx1 expression in thymocytes of Tg mice (A) and in transfected HeLa cell lysates used as NLS-hTrx1 protein control. Isolated thymocytes from Tg (6 mice) and WT (6 mice) were incubated with dexamethasone (Dex, 0, 1, 10 µM) for 4 h. Quantification of surviving cells in each group was measured 4 h after Dex treatment by WST-1 assay and confirmed by trypan blue cell counting. (Data are shown as means ± SE, 3 independent experiments, * p<0.05).

To determine whether increased nuclear Trx1 could similarly cause hyper-responsive immune signaling by another transcription factor with a redox-sensitive Cys in the DNA binding region, experiments were performed to examine glucocorticoid-stimulated apoptosis signaling in immature thymocytes. Dexamethasone (Dex) was used under conditions to cause limited (7%, 4 h) cell death in WT thymocytes measured by WST-1. Under identical conditions in Tg thymocytes (Fig. 8A), Dex caused significantly greater cell death (18%, Fig. 8B). Consequently, the results suggest that increased nuclear Trx1 could result in excessive signaling by GH and other transcription factors with redox-sensitive Cys residues in the DNA-binding regions.

## Discussion

Compartmentalized redox regulation is a key component of redox signaling and regulation of cell functions. Studies by Curran and coworkers showed that early stress response by AP-1 involved redox regulation in cell nuclei [9]. DNA binding was inhibited by oxidation of Fos and Jun, and Trx1 reversed this inhibition in the presence of NADPH, Trx1 reductase, and a nuclear redox factor (Ref-1). Ref-1 was found to be an activity of a DNA repair enzyme apurinic/apyrimidinic endonuclease [41]. Subsequent studies showed that similar nuclear redox control mechanisms function for NF-κB, Nrf-2, GR, estrogen receptor, p53 and HIF-1α [6], [8], [13], [16], [17], [42]. Importantly, Hirota et al. found that cytoplasmic Trx1 suppressed NF-κB activation by UV irradiation, whereas targeted expression of Trx1 in nuclei enhanced NF-κB activity by increasing DNA binding [5]. These results clearly discriminated nuclear and cytoplasmic redox events in transcriptional regulation. Site-directed mutagenesis suggested that lysine residues near the C-terminus of Trx1 provide a nuclear targeting sequence [43]. Together, the studies show that in response to oxidative stress, nuclear Trx1 functions to maintain redox-sensitive transcription factors in reduced, DNA-binding forms.

Nuclear Trx1 is relatively reduced under non-stressed conditions [44] and highly resistant to oxidation compared to cytoplasmic Trx1, mitochondrial Trx2 and cellular GSH [3]. Thus, there would not appear to be a need for nuclear translocation in the absence of oxidative challenge. None-the-less, Trx1 is found in nuclei of cells at growth boundaries [45]. This indicates that nuclear translocation of Trx1 occurs in response to signals other than overt oxidant stress. Nuclear targeting of Prx1, a Trx1-dependent peroxidase, enhances NF-κB reporter activity while cytoplasmic targeting of Prx1 blocks this activity [21], suggesting the existence of a constitutive H_2_O_2_-dependent mechanism to inhibit transcriptional activity. H_2_O_2_ production within nuclei occurs due to oxidative demethylation of histones [46], and stimulation of oxidant production appears to be a common event associated with DNA damage and repair [47]. Thus, the pattern of nuclear Trx1 in growth boundaries and the increase in response to stress may reflect temporal sequences in which transcriptional activation is followed by a nuclear oxidation that coordinately decreases activity of redox-sensitive transcription factors. Together, these observations suggest that nuclear Trx1 has dual functions in supporting activation of redox-sensitive transcription factors and also in inhibiting inactivation mechanisms that depend upon endogenous oxidants.

**Figure 9:**
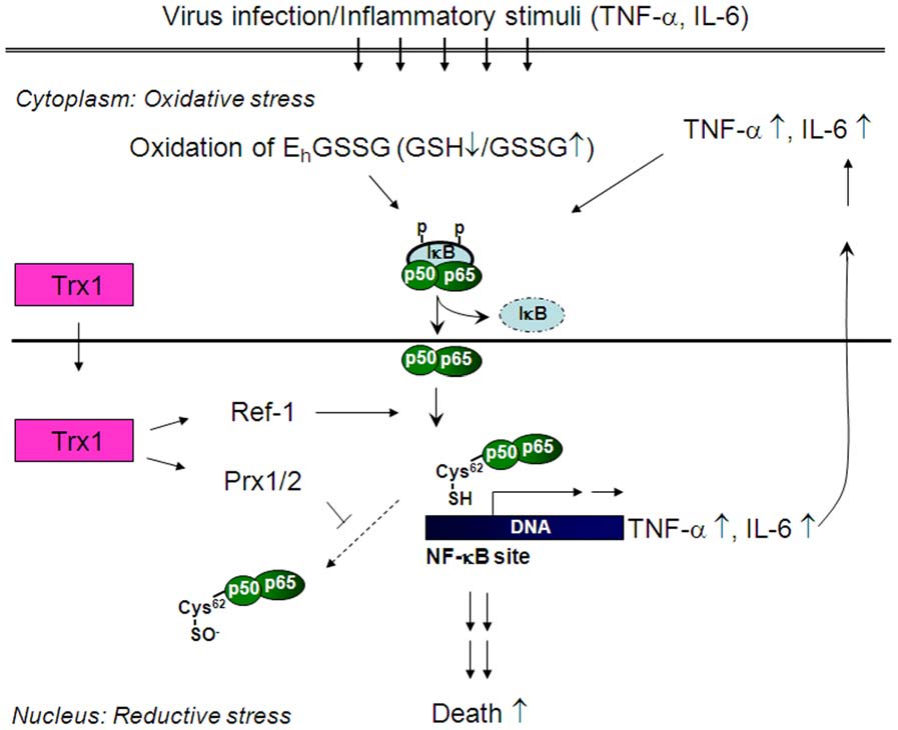
A scheme for stimulation of inflammatory response in NLS-hTrx1 Tg by infection. Virus infection and other inflammatory stimuli affect cytoplasmic redox state, e.g. oxidation of E_h_GSSG. Cytoplamic oxidation results in phosphorylation and degradation of I-κBα, which then translocates NF-κB to nucleus. Upon its translocation, p50 subunit (Cys62) reduced by nuclear localized Trx1 together with Ref-1 and Prx stimulates its DNA binding activity followed by subsequent gene expression, e.g. TNF-α and IL-6. Increased inflammatory cytokines further activate NF-κB as feedback stimulation.

A model incorporating these concepts to account for excessive immune response due to increased nuclear Trx1 is summarized in Fig. 9. The response to infection/inflammation includes: 1) decreased GSH levels by oxidative stress; 2) NF-κB activation and nuclear translocation; 3) elevation of NF-κB activity in nuclei due to increased abundance of Trx1 (NLS-hTrx1) and reduction via Ref-1; 4) increased cytokine gene expression by NF-κB and enhanced immune response; 5) decreased oxidative inactivation of NF-κB due to enhanced elimination of nuclear H_2_O_2_ by Trx1-dependent Prx-1 and Prx-2; 6) excessive stimulation of NF-κB activity; and 7) increased morbidity and mortality from excessive immune response. NF-κB activation is induced by viruses/viral products as well as other stimuli associated with oxidative stress (free radicals, UV light, gamma-irradiation) and could be generally relevant to conditions with increased signaling by inflammatory cytokines IL-6 [48] and TNFα [49]. Responses to infection-induced inflammatory stimuli include feedback stimulation, e.g. NF-κB activation stimulates inflammatory cytokine up regulations. The response is dependent upon GSH so the redox regulation may be more complex than implied by this model.

In the absence of viral challenge, the NLS-hTrx1 mice did not show gross phenotypic differences, e.g., weight, growth, fecundity, appearance, activity, from WT, and no major effects were observed in other thiol antioxidant proteins. This suggests that the present model can be useful to study oxidative processes that occur in cell nuclei. In this regard, the model adds to the growing number of mouse models useful to translate compartment-specific mechanistic information to *in vivo* function [50], [51]. This nuclear redox model may also be useful in combination with other compartmental redox models (e.g., Trx2 Tg mouse, [52], [53] to provide tools to help discriminate between toxicological stimuli targeting nuclear redox functions from those targeting cytoplasmic and mitochondrial functions. The HeLa cells studies (Fig. 5C), showed that increased NF-κB luciferase activity due to expression of NLS-hTrx1 (same vector as used for Tg mouse) was blocked by the dominant negative NLS-C35S-hTrx1. Consequently, development of mouse models with dominant negative Trx1 or an NF-κB reporter may enhance further understanding of redox control system in cell nuclei *in vivo*.

The exacerbation of injury with increased nuclear Trx1 is distinct from previous Trx1- and Trx2-transgenic mouse studies that showed protection against injury [39], [54], [55]. This distinct characteristic is reflected in the GSH data for lung and plasma (Fig. 7), which showed that the increased nuclear Trx1 did not affect cellular GSH or redox potential changes in response to infection but caused a significant downstream decrease in plasma GSH and oxidation of plasma E_h_GSSG. This is consistent with evidence that reductive stress, as well as oxidative stress, can contribute to disease mechanisms due to disruption of redox signaling and control. For instance, recent studies show involvement of reductive stress in cardiomyopathy [56], [57]. High levels of Hsp27 in transgenic mice resulted in cardiac hypertrophy in association with decreased ROS level, and significant increase in GSH content and the ratio of GSH/GSSG [57]. A consequence of reductive stress resulting from Hsp27 over-expression was a significantly reduced survival rate [57]. Rajasekaran et al. also demonstrated that cardiomyopathy induced by mutation in human αB-crystallin was under reductive stress due to elevated levels of glutathione peroxidase, increased GSH content and the ratio of GSH/GSSG and γ-glutamylcysteine synthetase [56].

Contribution of nuclear Trx1 to hyperactivity of the immune system could provide the basis for therapeutic development to inhibit nuclear Trx1 translocation as a means to prevent excessive activation following influenza infection. With some capacity to maintain reduction of transcription factors already present in nuclei, controlling further increase might limit intensity of activation without blocking necessary activity. Therapeutics targeting Trx1 translocation may also be useful for adult respiratory distress and multi-systems organ failure where post-infection immune responses contribute to tissue injury and death [58]. This approach could be useful to indirectly control stress-induced glucocorticoid responses, or in cancer therapeutics, provide a novel mechanism to control HIF-1α activity [59].

In summary, the present studies with H1N1 influenza viral infection in NLS-hTrx1 Tg mice show that increased nuclear Trx1 enhances activity of redox-sensitive transcription by NF-κB, causes exaggerated immune response and contributes to disease severity. Consistent with this response, redox-sensitive glucocorticoid-induced cell death was also exacerbated in thymocytes from Tg compared to WT littermates. Together, the results show that increased nuclear Trx1 has a critical role in stimulating intensity of immune responses. This suggests that nuclear translocation of Trx1 may be a useful therapeutic target to prevent severity of disease caused by excessive or prolonged activation of redox-sensitive transcription factors.
